# Factors affecting formation of adventitious branches in the seaweeds *Fucus vesiculosus* and *F. radicans*

**DOI:** 10.1186/s12898-019-0239-7

**Published:** 2019-06-04

**Authors:** Alexandra Kinnby, Ricardo T. Pereyra, Jonathan N. Havenhand, Pierre De Wit, Per R. Jonsson, Henrik Pavia, Kerstin Johannesson

**Affiliations:** Department of Marine Sciences, Tjärnö Marine Laboratory, University of Gothenburg, 452 96 Strömstad, Sweden

**Keywords:** Asexual reproduction, Baltic Sea, Macroalgae, North Sea, Salinity, Stress, Structural equation modelling

## Abstract

**Background:**

In the brackish Baltic Sea, shedding of adventitious branches is central to asexual recruitment of new thalli in the brown algae *Fucus vesiculosus* and *F. radicans*. To test which factors influence the formation of adventitious branches in brackish and in more marine conditions, we sampled 29 *Fucus* sites in the Baltic Sea (salinity 3–11) and 18 sites from the Danish straits, Kattegat, Skagerrak, and the North Sea (salinity 15–35). Separately for each area, we used structural equation modelling to determine which of eight predictor factors (phosphate, nitrate, chlorophyll-*a* (as a proxy for turbidity), temperature, salinity, oxygen, grazing pressure, and thallus area) best explained observed numbers of adventitious branches.

**Results:**

In more marine waters, high yearly average values of phosphate, salinity and turbidity had positive effects on the formation of adventitious branches. In brackish-waters, however, high numbers of adventitious branches were found in areas with low yearly average values of temperature, salinity and oxygen. Grazing intensity had no significant effect in either of the two study areas, contrasting findings from studies in other areas. In areas with both sexually and asexually reproducing *Fucus* individuals, clones had on average more adventitious branches than unique genotypes, although there was strong variation among clonal lineages.

**Conclusion:**

This study is the first to investigate multiple potential drivers of formation of adventitious branches in natural populations of *Fucus*. Our results suggest that several different factors synergistically and antagonistically affect the growth of adventitious branches in a complex way, and that the same factor (salinity) can have opposing effects in different areas.

**Electronic supplementary material:**

The online version of this article (10.1186/s12898-019-0239-7) contains supplementary material, which is available to authorized users.

## Background

**Fig. 1:**
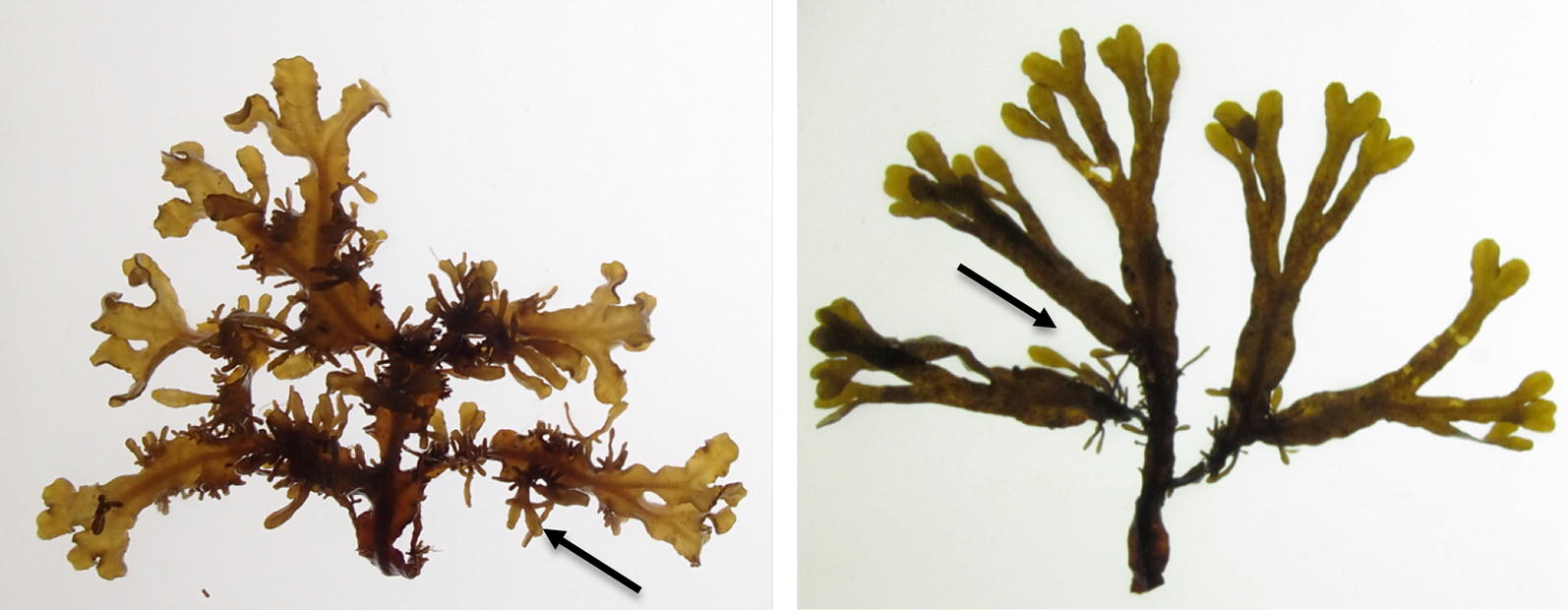
Individuals of *Fucus* spp. with many and few adventitious branches

Adventitious branches are new tissue grown from roots or stems in plants, or from the thallus in algae (Fig. 1). In terrestrial plants, adventitious branches are produced as a means of tissue repair and regeneration [1–3]. Growth of adventitious branches may be induced by grazing [4], but may also be induced by other stimuli, such as in trembling aspen (*Populus tremuloides*) where production of adventitious shoots on lateral roots is more affected by soil temperature (linked to fire) than by physical damage [5]. Additional stress factors reported to affect growth of adventitious branches in terrestrial plants include salt stress, hormone stress, light stress, removal of the apical bud, and chemical stress [6, 7].

In many marine macroalgae, adventitious branches are reported to develop as a way to heal wounds and regenerate tissue [8, 9]. In the fucoid brown alga *Fucus distichus*, Van Alstyne [10], showed that the mean number of adventitious branches was positively correlated with the abundance of herbivorous marine snails (*Littorina* spp.), suggesting that tissue repair may be an important factor. Experimental changes in water temperature and light exposure can induce growth of adventitious branches in the fucoid *Sargassum horneri* [11]. However, adventitious branches have also been observed to be generated in *F. distichus* without any obvious physical stimulus [10], highlighting that the control mechanism is complex.

*Fucus* spp. can form adventitious branches from both the thallus and the holdfast [10, 12, 13], and regeneration has been shown to be more common, and faster, from the midrib region of the thallus than from the thallus wings [12]. In many species of macroalgae, regeneration means apical growth of branched or un-branched filaments forming from the injury site. However, species of *Fucus* are atypical in this respect: epidermal cells divide perpendicularly to the surface and grow outwards, such that they develop into distinct ‘embryos’, as described by McCook and Chapman [14], instead of forming lateral branches [14]. These ‘embryos’ are morphologically indistinguishable from sexually produced embryos (sporelings) at early stages of development [12]. Although the adventitious branches resemble embryos, asexual reproduction of benthic thalli has not been observed in any *Fucus* species with one notable exception: in the Baltic Sea, detached adventitious branches produced by *F. vesiculosus* and *F. radicans* may reattach to the substratum by developing rhizoids, comparable to root hairs in vascular plants, and grow into new, seemingly functional male and female thalli, resulting in asexual recruitment of new individuals [15]. The reattachment of loose adventitious branches in Baltic Sea *Fucus* spp. has resulted in clones spread over extensive geographic areas (550 km) [16, 17].

It remains unclear what factors determine the incidence of asexual reproduction in *Fucus* species in the Baltic Sea. Experimentally decreasing salinity lead to the cessation of receptacle formation in three Finnish populations while vegetative growth largely remained intact [18]. In addition, polyspermy has a negative impact on sexual reproduction through reduced fertilization success below a salinity of 6–8, in the northern Baltic Sea [19, 20]. In any case, the formation of adventitious branches appears to be central to the evolution of asexual recruitment of the Baltic Sea *Fucus*.

We undertook an extensive mapping of the occurrence of adventitious branches in natural populations of *F. vesiculosus* and *F. radicans* inside and outside the Baltic Sea and used structural equation modeling to distinguish the effects of eight environmental factors, in order to investigate which factors promote the formation of adventitious branches in areas of different salinities. Based on prior knowledge that osmotic stress may induce formation of adventitious branches in vascular plants [6, 7], we hypothesized that salinity both inside and outside the Baltic Sea would have a strong effect on the prevalence of adventitious branches in fucoid seaweeds. Following an earlier report that adventitious branches correlate with grazer density [10], we also hypothesized that in areas with high grazing pressure, i.e. the more marine environment outside the Baltic Sea, tissue damage, measured as grazing marks, would promote formation of adventitious branches. In addition, we also included several other factors that may affect the formation of adventitious branches: temperature, oxygen, nitrate, phosphate, chlorophyll-*a*, and thallus area.

## Methods

### Study areas

The focus of this study was to distinguish which factors promote the formation of adventitious branches in *Fucus* spp. in sites located in two rather different environments: the Baltic Sea, and the transition zone (Danish straits, Kattegat, and Skagerack) and the eastern North Sea [21]. The two regions were separated with a line from Falsterbo in Sweden to Travemünde in Germany, following Johannesson and André [22] who found that for many taxa the area around this line separates two phylogeographic regions: the Baltic Sea and the eastern North Sea including the transition zone. Later studies have strongly corroborated this finding [23, 24].

In the Baltic Sea, the salinity (2–11) and surface water temperature decreases toward the north. Nutrient supply also declines from south to north, although the eastern Baltic Sea, including the Gulf of Finland, is nutrient-rich and eutrophic. The far north part, the Bay of Bothnia, is phosphorous-limited whereas the Baltic Sea proper is nitrogen-limited. The concentration of chlorophyll-*a* in the water column follows that of nutrients: high in the Gulf of Finland, the Gulf of Riga and in the Baltic Proper while lower in the Gulf of Bothnia as well as towards the Danish Straits [21]. The main *Fucus* herbivore in the Baltic Sea is the isopod *Idotea baltica* [25].

In the transition zone and the eastern North Sea the surface salinity increases and temperature decreases and stabilizes from south to north, from a salinity of 15 and variable temperature in the Danish Straits to a salinity of 35 and more stable temperatures in the eastern North Sea [21, 26]. A greater influence of deep, pelagic water from the Norwegian Trench lends a stabilizing influence in the north. Total dissolved nitrogen increases northwards in this area, while total dissolved phosphate and chlorophyll-*a* decreases, with the overall levels of dissolved phosphate being somewhat higher than in the Baltic region in general [26]. The *Fucus* grazing community in the transition zone and the eastern North Sea is dominated by littorinid snails and isopods, and is more diverse than in the Baltic Sea [25, 27, 28].

### *Fucus vesiculosus* and *Fucus radicans*

*Fucus radicans* is a very young species, endemic to the Baltic Sea, and formed by a recent split from the Baltic lineage of *F. vesiculosus* [29, 30]. Both species are dioecious (separate female and male individuals) and in the Baltic Sea, they reproduce both sexually and asexually through the formation of adventitious branches [15, 16] (Fig. 1). Due to their recent divergence, the morphological and/or genetic discrimination into separate species is still challenging in some areas of the Baltic Sea (see [17, 30]), including some localities from this study. We compared the number of adventitious branches between the two species in one sympatric site where they can be discriminated and found no significant difference (t-test, *t*_43_ = 0.36; p = 0.72). Hence, for this study’s purpose, we treated both taxonomic entities as *Fucus* spp.

### Sampling and image analysis

**Fig. 2:**
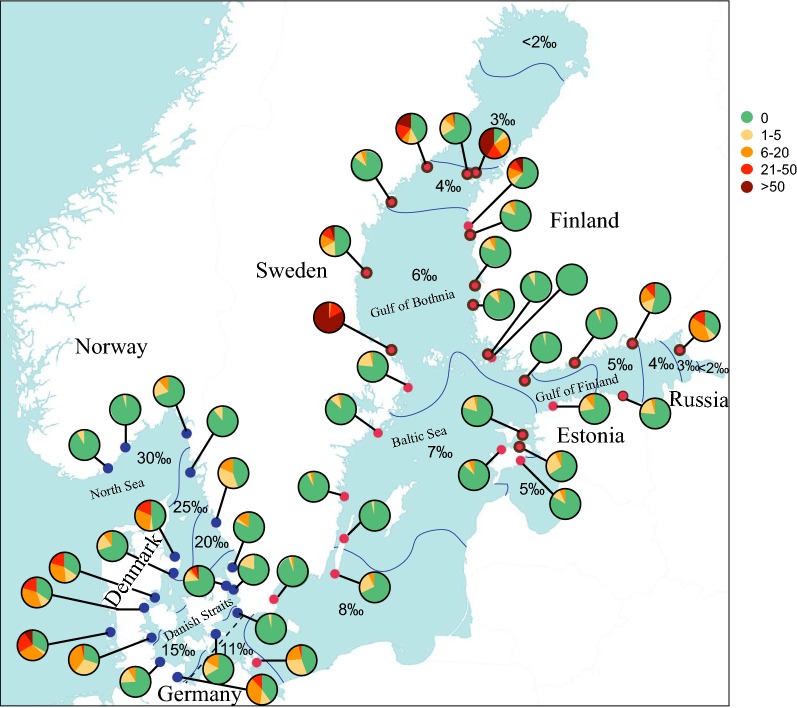
Map of the study area illustrating the salinity gradient from the eastern North Sea and into the Baltic Sea. Sampling sites from the transition zone and eastern North Sea are represented by blue (dark) circles and sites from the Baltic Sea are represented by red (light). Genotyped individuals within the Baltic Sea sites are represented by a black circle perimeter. The dashed line indicates the border between the Baltic Sea and the Danish Straits used in this study. Pie chart categories indicate the ratio of individuals in each population with a given number of adventitious branches, assembled into categories

Between 30 and 166 individuals of *Fucus* spp. were collected from each of 47 sites during September 2014 (Fig. 2). All individuals were photographed at the time of collection; small individuals were photographed in their entirety; larger individuals were subsampled by photographing one or a few large branches. The photographs were used to quantify the number of adventitious branches, and the surface area covered by the photo was estimated by image analysis in ImageJ [31].

Adventitious roots in terrestrial plants have been shown to form as a response to both mechanical and environmental stress [32, 33], including oxygen deprivation [34], and nutrient deficiency [33]. Studies looking at inducement of adventitious branches in algae are rare, but growth of adventitious branches in *Sargassum horneri*, (order fucales) is affected by temperature and light wavelength [11]. Thus, we conclude that a number of different abiotic variables may potentially affect growth of adventitious branches in *Fucus* spp., and we decided to explore five abiotic variables and three biotic variables (grazing pressure, thallus area, and chlorophyll-*a*) in our analysis. Data for salinity, temperature, nitrate, phosphate, oxygen, and chlorophyll-*a* (affecting light penetration and hereafter referred to as turbidity) for each site were taken from monitoring data from the ICES database (International Council for the Exploration of the Seas: data were mean values for the years 2005–2015). Monitoring sites were located 2.0–77.0 km (average 16.7 km) from the *Fucus* spp. sampling sites and sampled monthly to bimonthly. The majority of all *Fucus* spp. individuals included in our study showed no sign of grazing at all. Those that were grazed had clear grazing marks on the thallus but no major damage to the tissue, and so we assessed grazing pressure as presence or absence.

### DNA extraction and microsatellite genotyping

DNA was extracted from lyophilized fresh algal tissue using a CTAB modified protocol for genomic DNA [35]. Following this, samples were genotyped at five microsatellite loci shown to be diagnostic in previous studies of fucoid species [17, 36]. PCR products were pool-plexed and sized on a Beckman-Coulter CEQ 8000 capillary sequencer, and fragments were analyzed using the Fragment Analysis Software (Beckman-Coulter Inc., Fullerton, CA, USA). Genotypes were checked for null alleles, stuttering and allelic drop-out, using MICRO-CHECKER v. 2.2.3 [37]. GENCLONE 2.0 [38] was used to distinguish between unique genotypes and repeated genotypes produced by vegetative reproduction through reattachment of adventitious branches.

### Statistical analyses

#### Structural equation modelling

Structural equation models (SEM) were used to distinguish which factors affect the formation of adventitious branches. Separate structural equation models were used for individuals from the two sampling areas, the Baltic Sea (n = 1458), and eastern North Sea and the transition zone (n = 527) (Fig. 2). In addition, we also conducted analyses of a subset of the populations in the Baltic Sea where it was possible to unambiguously identify clones (n = 348) from unique genotypes (n = 443), i.e. asexually and sexually derived individuals, respectively. Within the clonal subgroup one very dominant genotype (183 individuals of the same clone) was excluded.

Our primary goal was to find out which factors affect formation of adventitious branches in our two study regions, and we therefore studied the number of adventitious branches in relation to several parameters: temperature, salinity, phosphate, nitrate, turbidity, oxygen, thallus area, and grazing pressure. Structural equation modeling makes it possible to separate the effect of each factor into direct, indirect, and total (the sum of direct and indirect) effects. Factors with strong indirect effects exert their influence by having strong effects on other factors in the analysis. We focused on factors that had either a strong direct or a strong total effect on the number of adventitious branches.

Before SEM analysis, all predictor variables were standardised and tested for multi-collinearity by calculating the correlation coefficient for all possible combinations between the factors, and the variance inflation factor (VIF) was calculated for each predictor. All correlation coefficients were below 0.7 and all VIFs less than 2, indicating that they do not exhibit collinearity [39]. A theoretical model was constructed based on these results and previous knowledge. From this starting point, non-significant paths were removed one at a time, starting with the highest p-value pathway. Modification indices were used as a guide to add missing paths, but only where those paths were ecologically reasonable. These steps were repeated until further changes did not affect the significance of the χ^2^ value. We report the model with the lowest Akaike Information Criterion (AIC) and good fit indices (Table 1).

**Table 1:** SEM statistics

Model	df	χ^2^	p	CFI	TLI	RMSEA	SRMR
Baltic Sea	8	13.055	0.110	0.99*	0.99*	0.021*	0.013*
Transition zone and eastern North Sea	7	11.18	0.131	0.99*	0.99*	0.034*	0.020*
Genetically unique genotypes	5	7.443	0.190	0.99*	0.99*	0.033*	0.010*
Clones	4	6.354	0.174	0.99*	0.99*	0.041*	0.019*

Fit indices for the four structural equation models. For each model we report five fit indices: Chi-square, *CFI* the comparative fit index, *TLI* Tucker-Lewis index, *RMSEA* the root mean square error of approximation, *SRMR* standardized root-mean-square residual, using the following cut-off values for a ‘good fit’: CFI ≥ 0.95, TLI ≥ 0.95, RMSEA ≤ 0.08, SRMR ≤ 0.06 [31, 40–42]

*Denotes significant values

For each structural equation model we report five fit indices with respective cut-off values for a ‘good fit’: Chi-square (> 0.05), CFI (the comparative fit index; CFI ≥ 0.95) [40]; TLI (Tucker-Lewis index; TLI ≥ 0.95) [41]; RMSEA (the root mean square error of approximation; RMSEA ≤ 0.08 [42]; and SRMR (standardized root-mean-square residual; SRMR ≤ 0.06) [40].

Structural Equation Models were run in RStudio (version 1.0.136) using the *lavaan* package [43]. Path diagrams were generated with the package *SemPlot* [44].

#### Comparison between clones and genetically unique individuals

A Welch’s *t*-test was used in RStudio (version 1.0.136) to investigate whether clonal individuals (n = 531) had more adventitious branches than genetically unique individuals (n = 443). Welch’s *t*-test was chosen since homogeneity of variance could not be assumed [45].

## Results

The prevalence, as well as number, of adventitious branches varied between geographical areas, with the highest prevalence in the south west (Danish coast) and in the north east (northern Swedish and Finnish coasts) (Fig. 2). Additionally, the innermost sites of the Gulf of Finland also had high prevalence of adventitious branches (Fig. 2).

**Fig. 3:**
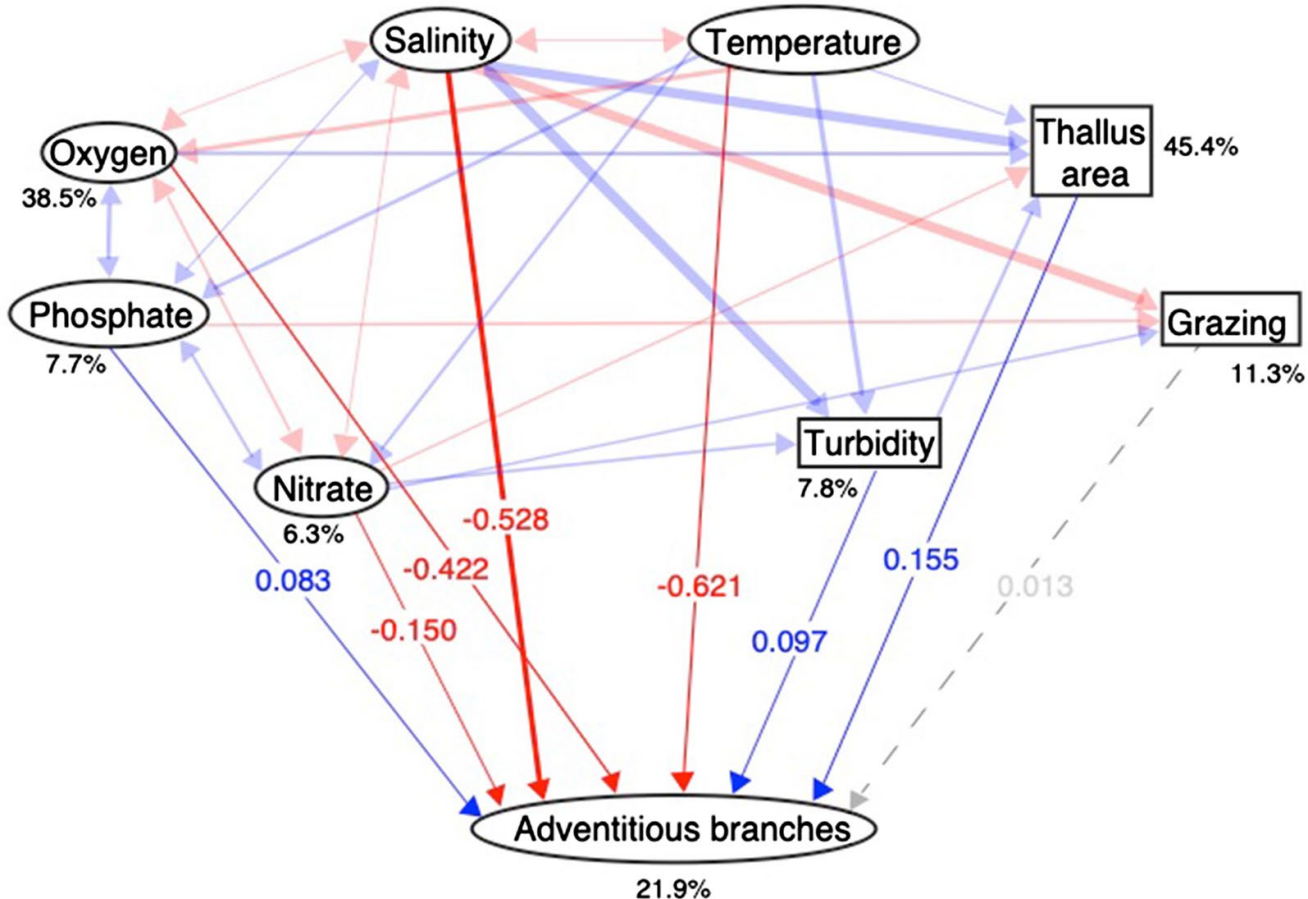
Path diagram based on structural equation modelling showing how the number of adventitious branches per thallus in Baltic Sea *Fucus* spp. populations is affected by different environmental factors. Red arrows indicate negative path coefficients, blue arrows indicate positive path coefficients, and dashed gray arrows indicate non-significant paths. All path coefficients are standardized. Variables presented in rectangles are biotic while those presented in ovals are abiotic (with the exception of adventitious branches). Percentages indicate the variance explained by the model, all correlation coefficients are low (p < 0.00001)

For all structural equation models performed, fit indices indicated that the observed data did not differ statistically from the model, indicating a good model fit (Table 1). Within the Baltic Sea, temperature, salinity, oxygen, and nitrate all had negative total effects on the number of adventitious branches, with temperature, salinity and oxygen generating the strongest total effects (Fig. 3, Table 2). Positive, but weaker, effects were observed for increased thallus area, turbidity, and phosphate (Fig. 3, Table 2). Notably, grazing had a non-significant effect on the number of adventitious branches in *Fucus* spp. from the Baltic Sea.

**Table 2:** Estimated standardized total effects (i.e. the sum of the direct effect and all possible indirect effects) and direct effects with corresponding p-values from the structural equation model for *Fucus* spp. populations located in the Baltic Sea

	Direct effect	p-value (direct)	Total effect
Thallus area (cm^2^)	0.155	0.000	0.155
Turbidity	0.097	0.003	0.177
Grazing	0.013	0.601	0.013
Nitrate (µmol/l)	− 0.150	0.000	− 0.157
Oxygen (ml/l)	− 0.422	0.000	− 0.355
Phosphate (µmol/l)	0.083	0.006	0.080
Salinity	− 0.528	0.000	− 0.468
Temperature (°C)	− 0.621	0.000	− 0.351

Positive denotes an increase in the number of adventitious branches

**Fig. 4:**
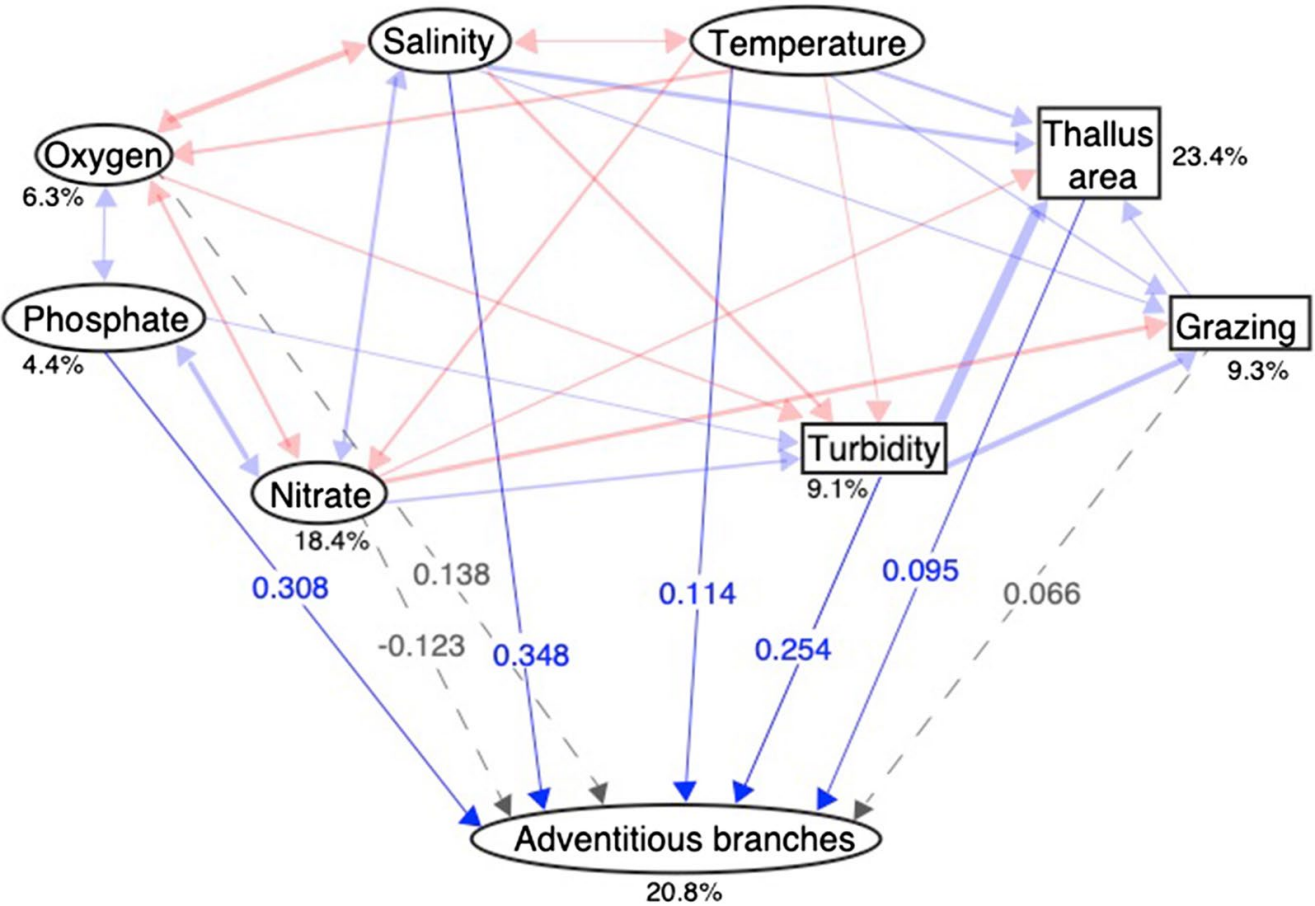
Path diagram based on structural equation modelling showing how the number of adventitious branches per thallus in *Fucus* spp. populations from the transition zone and eastern North Sea is affected by different environmental factors. Red arrows indicate negative path coefficients, blue arrows indicate positive path coefficients, and dashed gray arrows indicate non-significant paths. All path coefficients are standardized. Variables presented in rectangles are biotic while those presented in ovals are abiotic (with the exception of adventitious branches). Percentages indicate the variance explained by the model, all correlation coefficients are low (p < 0.00001 except nitrate which is low significant p = 0.000114)

Structural equation model analysis of the transition zone and eastern North Sea sites revealed that high levels of salinity, phosphate, turbidity, temperature, and thallus area had significant positive direct effects and positive total effects on the number of adventitious branches per thallus in this area (Fig. 4, Table 3). This was in marked contrast to the results for the Baltic Sea, where, in particular, increased salinity had the opposite effect. Oxygen, nitrogen, and grazing all showed non-significant effects in the transition zone and eastern North Sea (Fig. 4, Table 3).

**Table 3:** Estimated standardized total effects (i.e. the sum of the direct effect and all possible indirect effects) and direct effects with corresponding p-values from the structural equation model for *Fucus* spp. populations located in the transition zone and the eastern North Sea

	Direct effect	p-value (direct)	Total effect
Thallus area (cm^2^)	0.095	0.032	0.095
Turbidity	0.254	0.000	0.313
Grazing	0.066	0.107	0.075
Nitrate (µmol/l)	− 0.123	0.187	− 0.145
Oxygen (ml/l)	0.138	0.094	0.158
Phosphate (µmol/l)	0.308	0.000	0.308
Salinity	0.348	0.001	0.161
Temperature (°C)	0.114	0.026	0.078

Positive denotes an increase in the number of adventitious branches

The structural equation models for both clones and genetically unique individuals from the northern Baltic Sea demonstrated a similar pattern to that seen for the whole Baltic Sea. In genetically unique individuals, increasing temperature, oxygen, and salinity had negative direct effects (i.e. fewer adventitious branches), while increased levels of phosphate instead showed a positive direct effect (i.e. more adventitious branches). Thallus area, turbidity, nitrate and grazing all had non-significant effects (Additional file 1: Figure A1, Table A1). In clonal individuals, higher temperature, oxygen, nitrate, and salinity all showed negative direct effects on number of adventitious branches, while increased phosphate and thallus area generated positive direct effects. Turbidity and grazing did not show any significant effects among the clonal thalli (Additional file 1: Figure A2, Table A2).

Average number of adventitious branches was much higher in clonal individuals (mean = 26.0; 95% CI 21.7–28.4) than in genetically unique individuals (mean = 4.0; 95% CI 1.6–4.5) (Welch’s *t*-test, *t*_719_= 11.86; p < 0.0001).

## Discussion

In the Baltic Sea, low salinity, temperature, and oxygen were all associated with high number of adventitious branches, suggesting that increased physical stress promotes formation of adventitious branches. Outside the Baltic Sea the pattern was very different. High levels of salinity together with increased availability of phosphate and high turbidity were associated with increased number of adventitious branches. In general, the effects of environmental factors were weaker in the marine environment than in the brackish Baltic Sea.

The positive effects of phosphate and turbidity on the number of adventitious branches observed in the more marine regions were linked to phytoplankton blooms decreasing light penetration [46] and imposing light-stress on fucoid thalli. Such a stress might induce increased formation of adventitious branches. For example, it has been shown in some macroalgae that light intensity and wavelength can play a role in formation of adventitious tissue. Uji et al. [11] found that *Sargassum horneri* cultures grown under white and blue light developed more adventitious tissue than those cultured under red light; broadly equivalent responses were seen in Laminariales [47], but not in higher plants [48]. There are too few data to draw definitive conclusions; however, it is interesting to note that *Fucus* spp. grow exclusively subtidally (and at greater depths) in the Baltic Sea than in the transition zone and eastern North Sea [49].

The hypothesis that grazing induces production of adventitious branches in fucoids was proposed as early as the nineteenth century (Oltmanns 1889 from [50]). This hypothesis has since been supported by observations of a correlation between adventitious branch production and grazer (*Littorina* spp.) density, as well as by demonstrated grazing preferences associated with polyphenolic compound content of adventitious branches relative to ‘normal meristematic tissue’ from apices and thalli [10]. In the present study, however, we did not find a relationship between grazing intensity and adventitious branch formation in either of the two regions, despite the fact that grazing can be intense both in the southern parts of the Baltic Sea and in the marine region [28].

In both study regions, most direct effects on the formation of adventitious branches were a small fraction of the total effects (Tables 2, 3), indicating the importance of indirect effects of biotic and abiotic drivers. A singular exception to this pattern was present in the Baltic, however, where the direct (negative) effect of salinity on adventitious branches was approximately equal to the overall total effect. Nonetheless, total effect of salinity on adventitious branches in the Baltic Sea were less than those of temperature, once again indicating the importance of indirect effects. Outside the Baltic Sea, in the transition zone and eastern North Sea, effects were generally weaker, and patterns more complex, which is reflected in proportionally greater indirect effects (*cf* difference between direct effect and total effect in Tables 2, 3). Perhaps, formation of adventitious branches is more related to physical stress in the Baltic Sea *Fucus* spp., a region that was recently invaded by *Fucus* spp. and where it lives close to its range margin. In contrast, physical stress from the environment affecting production of adventitious branches may be relaxed in a more marine habitat, although North Sea *Fucus* spp. is more regularly exposed to environmental fluctuations as it inhabits the intertidal zone while it lives subtidally in the Baltic Sea [49].

The variables which exerted the strongest effect on the number of adventitious branches were the same for both clones and unique genotypes in the area where these co-existed (the northern Baltic Sea), but the effect strength (both direct and indirect) was much higher on clonal individuals. In addition, individual thalli of clones had on average more adventitious branches than those of unique genotypes, which we interpret as a consequence of selection favoring adventitious branch formation among asexual lineages to increase vegetative recruitment among asexual lineages. In both the Baltic Sea and the transition zone and eastern North Sea the models explained about 20% of the observed variation in number of adventitious branches. We here argue that the contribution of genetically-derived phenotypic variation to the remaining 80% unexplained variation is important. The reason we believe so is that when we tested the effects of environmental drivers on the number of adventitious branches in clonal individuals (effectively increasing the number of environmental replicates per genotype, Additional file 1: Figure A2) we found these explained 62% of the variation in number of adventitious branches. This strongly supports the suggestion that genetic factors contributed to the unexplained variance in the Baltic Sea and transition zone. This result also corroborates earlier observations of genetic variation among clones in number of adventitious branches [51].

## Conclusion

We show that the number of adventitious branches in natural populations of *Fucus* spp. are strongly affected by several environmental factors, in particular salinity, and that the effects of these factors differ between sites inside and outside the Baltic Sea: inside, the number of adventitious branches increased with declining salinity, whereas the opposite relationship was seen outside. Experimental work is needed to determine whether the environmental factors induce adventitious branch formation through a plastic response, or if they select for individuals that are genetically disposed to generate more adventitious branches. In addition, we suggest that variation among populations may be due to genetic differences resulting either from local adaptation uncorrelated to the physical factors we measured, or from stochastic effects of population separation. Interestingly, the formation of adventitious branches is a general and ancestral feature of marine populations of fucoid species (e.g. [8, 12, 52]), but in the Baltic Sea it has been a key component in the asexual recruitment and colonization of this brackish water environment. Identifying the environmental factors that drive formation of adventitious branches is an important step towards an increased understanding of how and why asexual reproduction is predominant in large parts of the Baltic Sea.

## Additional file

**Additional file 1.** Tables and figures containing results from structural equation models for genetically unique and cloned individuals of *Fucus* spp. from the Baltic Sea, respectively.

## Data Availability

The dataset analysed in the current study is available from the corresponding author on reasonable request.
