# Supplementary material for: Factors affecting formation of adventitious branches in the seaweeds Fucus vesiculosus and F. radicans
Source: BMC Ecol. 2019 Jun 4;19:22. doi: 10.1186/s12898-019-0239-7 (PMC6549257; doi:10.1186/s12898-019-0239-7)
Supplement: Supplementary file 1 — Additional file 1. Tables and figures containing results from structural equation models for genetically unique and cloned individuals of Fucus spp. from the Baltic Sea, respectively. [file 12898_2019_239_MOESM1_ESM.docx]

**Additional file 1**

**Table A1**. Estimated standardized total effects (*i.e.* the sum of the direct effect and all possible indirect effects) and direct effects with corresponding p-values from the structural equation model for genetically unique individuals of *Fucus* spp. populations from the Baltic Sea. Positive denotes an increase in the number of adventitious branches.

|  | **Direct effect** | **p-value (direct)** | **Total effect** |
| --- | --- | --- | --- |
| Thallus area (cm^2^) | 0.010 | 0.849 | 0.010 |
| Turbidity | -0.081 | 0.182 | -0.063 |
| Grazing | 0.047 | 0.444 | 0.047 |
| Nitrate (µmol/l) | -0.109 | 0.191 | -0.109 |
| Oxygen (ml/l) | -0.461 | 0.000 | -0.450 |
| Phosphate (µmol/l) | 0.211 | 0.011 | 0.160 |
| Salinity | -0.408 | 0.000 | -0.433 |
| Temperature (°C) | -0.510 | 0.000 | -0.247 |

Kinnby *et al*. Additional file 1

**Table A2**. Estimated standardized total effects (*i.e.* the sum of the direct effect and all possible indirect effects) and direct effects with corresponding p-values from the structural equation model for cloned individuals of *Fucus* spp. populations from the Baltic Sea. Positive denotes an increase in the number of adventitious branches.

|  | **Direct effect** | **p-value (direct)** | **Total effect** |
| --- | --- | --- | --- |
| Thallus area (cm^2^) | 0.410 | 0.000 | 0.410 |
| Turbidity | -0.117 | 0.064 | -0.047 |
| Grazing | -0.006 | 0.872 | -0.006 |
| Nitrate (µmol/l) | -0.476 | 0.000 | -0.620 |
| Oxygen (ml/l) | -1.005 | 0.000 | -0.784 |
| Phosphate (µmol/l) | 0.769 | 0.006 | 0.772 |
| Salinity | -0.434 | 0.000 | -0.346 |
| Temperature (°C) | -1.346 | 0.000 | -0.373 |

Kinnby *et al*. Additional file 1

**
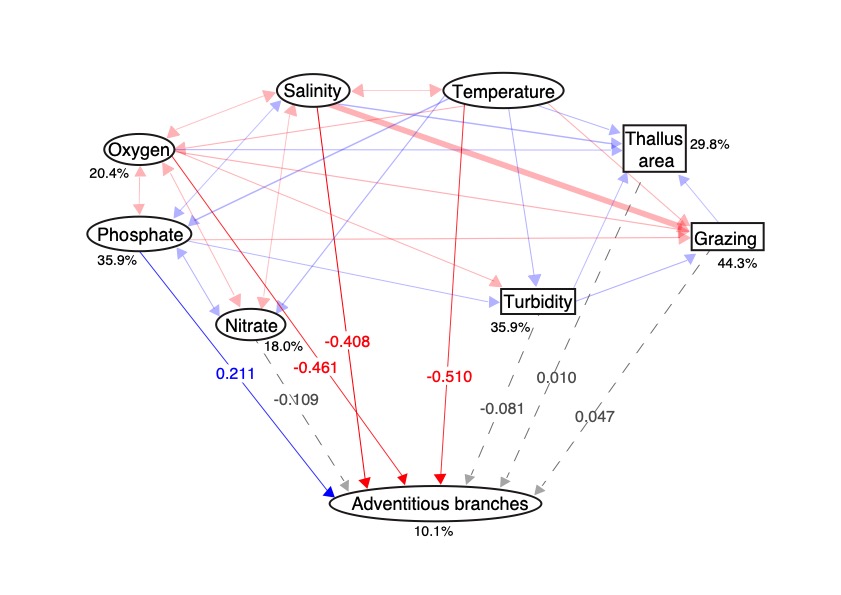
**

**Figure A1.** Path diagram based on structural equation modelling showing how the number of adventitious branches per thallus in individuals with unique genotypes of *Fucus* spp. populations from the Baltic Sea is affected by different environmental factors. Red arrows indicate negative path coefficients, blue arrows indicate positive path coefficients, and dashed gray arrows indicate non-significant paths. All path coefficients are standardized. Variables presented in rectangles are biotic while those presented in ovals are abiotic (with the exception of Adventitious branches). Percentages indicate the variance explained by the model, all correlation coefficients are low (p < 0.00001).

Kinnby *et al*. Additional file 1


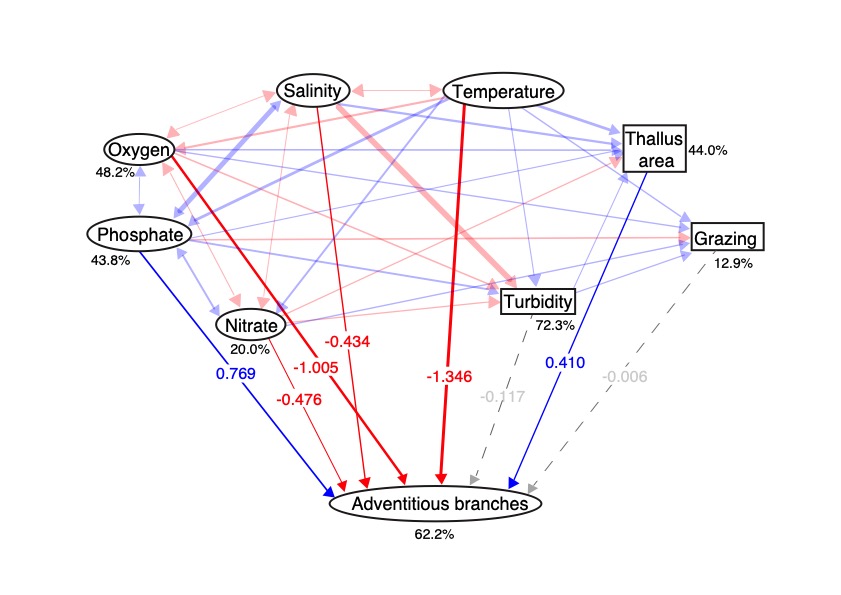
 **Figure A2.** Path diagram based on structural equation modelling showing how the number of adventitious branches per thallus in cloned, *i.e.* asexually reproduced individuals, of *Fucus* spp. populations from the Baltic Sea is affected by different environmental factors. Red arrows indicate negative path coefficients, blue arrows indicate positive path coefficients, and dashed gray arrows indicate non-significant paths. All path coefficients are standardized. Variables presented in rectangles are biotic while those presented in ovals are abiotic (with the exception of Adventitious branches). Percentages indicate the variance explained by the model, all correlation coefficients are low (p < 0.00001).

Kinnby *et al*. Additional file 1

**Figure A3.** Correlation plot of the number of adventitious branches per algae against thallus area for individuals of *Fucus* spp. from the Baltic Sea (n=1458).

Kinnby *et al*. Additional file 1

**Figure A4.** Correlation plot of the number of adventitious branches per algae against thallus area for individuals of *Fucus* spp. from the transition zone and eastern North Sea (n=527).

Kinnby *et al*. Additional file 1
